# General principles of minimally invasive surgery in paediatric surgical oncology

**DOI:** 10.3332/ecancer.2023

**Published:** 2025-11-13

**Authors:** Israel Fernandez-Pineda, Simone Abib

**Affiliations:** 1 Department of Paediatric Surgery, Children's Health Ireland at Our Lady's Children's Hospital, Dublin D12 N512, Ireland; 2 Department of Paediatric Surgery, Paediatric Oncology Institute – GRAACC, Federal University of São Paulo, São Paulo 04039-001, Brazil

**Keywords:** paediatric surgical oncology, minimally invasive surgery

## Abstract

Minimally invasive surgery (MIS) has become increasingly integrated into Paediatric Surgical Oncology (PSO), offering benefits such as faster recovery, reduced postoperative pain, earlier resumption of adjuvant therapy, lower blood loss and improved cosmetic outcomes. Despite these advantages, the safe application of MIS in oncology requires strict adherence to oncological principles to avoid complications such as tumour spillage, incomplete resections and staging errors, which may compromise survival outcomes. This article reviews the general principles, indications and contraindications for MIS in paediatric oncology, highlighting tumour- and histology-specific considerations. Commonly accepted MIS applications include selected cases of neuroblastoma, Wilms tumour following neoadjuvant therapy under SIOP protocols, thoracoscopic lung metastasectomy and resection of certain mediastinal, hepatic and adnexal masses. Contraindications include large or fragile tumours, high-risk neuroblastomas with vascular encasement and situations where surgeon experience or resources are insufficient. Technical aspects, patient selection and multidisciplinary coordination are emphasised as key to ensuring safety and efficacy. Establishing MIS guidelines in PSO may aid surgeons in decision-making and promote consistent standards of care.

## Introduction

Minimally invasive surgery (MIS) has evolved over the last decades, resulting in the preferred approach for certain conditions in children such as appendicitis, cholecystitis, pyloric stenosis, splenectomy and so on. The relatively low volume of Paediatric Surgical Oncology (PSO) cases encountered by general paediatric surgeons can negatively impact their decisions or confidence to use an MIS approach. MIS approach should be considered when the surgeon has proficiency in both open oncological procedures and MIS techniques; otherwise, important oncological principles violations may occur and impact the chances of cure and survival of children with cancer.

Historically, cancer surgery has been associated with big incisions and aggressive resections, which is still nowadays of great value for certain histologies, including high-risk neuroblastomas, sarcomas, locally advanced Wilms tumours, pulmonary metastatic disease from osteosarcoma and so on.

MIS in paediatric oncology remains an evolving field that has grown over the past 40 years. For MIS to gain wider acceptance, it must be able to at least replicate or exceed outcomes that are achieved with the current standard of care [1, 2]. With the advancement of MIS, paediatric surgical oncologists have turned their efforts to utilise these techniques for the benefit of children with cancer. Advantages of MIS for the paediatric cancer patients include early postoperative recovery, decreased postoperative pain, early re-initiation of adjuvant therapy (chemotherapy, radiation therapy, immunotherapy and stem cell transplantation), decreased intraoperative blood loss and improved cosmetic result [3]. From a technical standpoint, MIS provides magnification of the local anatomy, which refines the assessment of the limits of resection and a better visualisation for certain anatomic locations such as thoracic outlet, mediastinum and pelvis. Limitations of MIS in PSO include loss of tactile feedback, risk of vascular injury, limited working space when resecting large tumours, risk of tumour spillage and challenges related to removal of the resected specimen.

As a rule, if a MIS procedure is performed in a safe manner and following the same oncological principles used in open surgery, this should be promoted [4]. The reality is that MIS is associated with longer learning curves, which exposes patients to long procedures and is occasionally performed by paediatric surgeons who are not familiarised with the surgical guidelines of the cancer protocols. This may result in incomplete surgical resections, failures in local staging, tumour spillage and eventually local and systemic recurrences that may impact the long-term survival rates. These MIS guidelines may represent a helpful tool to clearly establish the indications and contraindications of a less invasive surgical approach for paediatric cancer patients. The guidelines also provide practical surgical tips to avoid complications and guide the surgeon through the procedure.

## Indications

The prognostic significance of radical resection and inherent technical complexity to achieve complete resection are both dependent on tumour and histology. There are certain MIS procedures in PSO that have gained popularity over the last years including low and intermediate risk neuroblastoma depending on the presence and nature of image-defined risk factors, radical nephrectomy for some renal tumours after neoadjuvant chemotherapy in the International Society of Paediatric Oncology (SIOP) protocol, thoracoscopic lung metastasectomy, biopsy and/or resection of mediastinal masses, liver masses, certain pancreatic tumours with a favourable anatomic location and resection of adnexal masses (Table 1) [5–8].

Thoracoscopic resection of lung nodules or mediastinal masses for both diagnostic and therapeutic purposes represents a good approach to avoid a thoracotomy [9]. The use of thoracoscopy is influenced by the tumour type. Patients with non-chemosensitive tumours such as osteosarcoma and nonrhabdomyosarcoma soft tissue sarcomas may benefit from aggressive attempts to remove even a single nodule and thoracotomy is usually recommended. Nevertheless, patients with oligometastatic pulmonary metastatic may be good candidates for the MIS approach.

Patients with lung metastatic disease in the context of chemosensitive tumours such as Wilms tumour, hepatoblastoma, germ cell tumours and rhabdomyosarcoma may benefit from thoracoscopic resections.

Localisation of lung lesions is usually required for subpleural nodules that are not visible on the lung surface. Although percutaneous biopsies may be performed with the assistance of image-guided technology, there remain situations in which a surgical biopsy is required either via thoracoscopic or open surgical technique. A limited thoracotomy incision or potentially a traditional posterolateral thoracotomy, which requires a large incision, rib retraction and possible division of the latissimus dorsi may be required to obtain adequate specimens. This morbid incision has led to long-term complications in children such as shoulder elevation, winged scapula, chest wall asymmetry and scoliosis; therefore, consideration for a thoracoscopic approach is certainly deserving in this special population [10, 11]. Thoracoscopic resection of neuroblastoma has been associated with a decreased hospital stay, less intraoperative blood loss and less requirements for chest drains.

A localised <5 cm adrenal mass is usually a good case for MIS, but if a suspicion for adrenocortical carcinoma (ACC) is raised, MIS should be discouraged. ACC is a poor chemo/radiation sensitive tumour; therefore, complete resection with negative margins is critical for long-term survival. ACC is generally a fragile tumour and at significantly high risk of rupture, which negatively affects survival and so an open approach is recommended to minimise this risk [12, 13].

Surgical management of Wilms tumour in North America is based on the Children’s Oncology Group (COG) guidelines and upfront resection is recommended. These tumours are generally large, fragile and at risk of rupture; therefore, MIS does not play a role in COG guidelines. On the other hand, the SIOP protocol recommends neoadjuvant chemotherapy for suspected Wilms tumours and MIS tumour nephrectomy may be feasible after chemotherapy-induced shrinkage. SIOP 2001 trial outcomes for patients with unilateral Wilms tumour who underwent MIS resection were comparable to open surgery; however, lymph node sampling was deficient in this study, which may impact the recurrence rate. Adequate lymph node sampling is mandatory for adequate local staging [7]. Nephron-sparing surgery (NSS) is frequently required in bilateral Wilms tumours. Laparoscopic NSS for malignant renal tumours remains controversial. There are potential challenges such as obtaining a negative resection margin, avoidance of tumour spillage and potentially increased chance of recurrence. It is technically very demanding and it should be performed by a very experienced team.

MIS for adnexal masses has an important diagnostic and staging role in malignant ovarian tumours; however, the risk of spillage limits the appropriateness of laparoscopic resection. For benign ovarian germ cell tumours such as teratoma, ovarian sparing surgery is recommended, but MIS tools are less able to delineate the interface between tumour and normal ovarian tissue; therefore, an open approach should be favoured over MIS if lack of experience [14].

## Contraindications

Large masses can potentially impede safe accessibility and specimen delivery, and contribute to the potential risk of intra-operative tumour spillage; therefore, tumour size should be considered when selecting the optimal surgical approach.

For certain thoracoscopic procedures, single lung ventilation is critical to obtain lung collapse and this is sometimes difficult to achieve in small patients. This can hamper visualisation of pulmonary nodules, decrease working space in the thorax and increase risk of lung injury. Therefore, patient selection plays a very important role in the success of the MIS procedure. Deep and small pulmonary nodules not amenable to localisation are difficult to visualise and since the tactile ability is lost in thoracoscopy, these pulmonary lesions may be missed and an open approach may be preferred. Osteosarcoma patients with multiple serial pulmonary metastasectomies are expected to have firm lung adhesions to the chest wall, which may limit the ability for a thoracoscopic approach.

High-risk neuroblastoma usually presents with midline involvement and encasement of major vascular structures, including aorta, cava, celiac axis, superior mesenteric artery and renal vessels. The extent of resection may affect the outcome of high-risk neuroblastoma patients; therefore, an open approach is generally recommended.

Other contraindications to perform MIS in paediatric oncology include inadequate equipment, insufficient training, lack of experience and patient-related factors such as tumour size, abnormalities in cardiac output, patient instability and coagulopathy [15].

## Surgical approach

Recent publications have shown that low complication and conversion rates of MIS tumour resection may be achieved with careful patient selection [16]. Other factors influencing the outcomes include the surgeon’s experience, patient size, location and proximity to vital structures. All these factors impact the final decision of whether or not to pursue this minimally invasive approach. Close communication with anaesthesia team is critical to maximise the safety of the procedure. MIS requires the ability to create enough working space to safely visualise and perform the operative procedure, which is obtained by means of carbon dioxide insufflation.

This may lead to difficulty with anesthesia in infants and young children. Other factors influencing the anesthetic procedure include single lung ventilation, hypothermia and the effect of lateral decubitus positioning for thoracoscopic procedures.

Technical factors influencing the success of MIS in PSO include right trocar placement, camera with zoom magnification, meticulous haemostasis, avoidance of heat dispersion, use of Endo-catch bags for tumour extraction and enlargement of trocar site.

## Conclusion

MIS in PSO is considered a safe diagnostic and therapeutic modality. Careful patient selection and correct surgical indications are critical to ensure oncologic principles are not violated and to minimise the potential for complications. It is important for the paediatric surgeon, as a member of the multidisciplinary team involved in the care of children with cancer, to understand the indications and contraindications of MIS in the treatment of paediatric solid tumours. We are optimistic that the creation of these guidelines for the use of MIS in PSO will help paediatric surgeons in the decision-making process for the best possible surgical approach.

## Conflicts of interest

The authors have no conflicts of interest to declare.

## Funding

This research received no specific grant from any funding agency in the public, commercial or not-for-profit sectors.

## Figures and Tables

**Table 1.** MIS indications for PSO according to anatomic location.

Laparoscopic	Neurogenic tumour (mainly adrenalectomy and resection of tumours without IDRFs). Avoid for ACC.
	Wilms tumour after neoadjuvant chemotherapy (SIOP protocol_certain cases)
	Liver tumour (diagnostic or therapeutic)
	Splenectomy for splenic malignant infiltrate and splenomegaly
	Oophoropexy prior to pelvic irradiation
	Pelvic mass
	Pancreatic tumour
Thoracoscopic	Lung nodule
	Neurogenic tumour without IDRFs
	Mediastinal mass (diagnostic or therapeutic)

ACC: adrenocortical carcinoma; IDRFs: image-defined risk factors; SIOP: International Society of Paediatric Oncology
